# Special Section Guest Editorial: Wearable, Implantable, Mobile, and Remote Biomedical Optics and Photonics

**DOI:** 10.1117/1.JBO.26.6.062701

**Published:** 2021-06-30

**Authors:** Jessica Ramella-Roman, Amir H. Gandjbakhche, Stephen C. Kanick, Babak Shadgan, Bruce J. Tromberg

**Affiliations:** a Florida International University, United States; b National Institute of Child Health and Human Development, United States; c Profusa Inc., United States; d University of British Columbia, Canada; e National Institute of Child Health and Human Development, United States

## Abstract

Guest editors Jessica Ramella-Roman, Amir H. Gandjbakhche, Stephen C. Kanick, Babak Shadgan, and Bruce J. Tromberg introduce and summarize the articles included in the 6-part JBO Special Section on Wearable, Implantable, Mobile, and Remote Biomedical Optics Photonics.

We are pleased to introduce this JBO Special Section entitled “Wearable, Implantable, Mobile, and Remote Biomedical Optics & Photonics,” published as a 6-part series from August 2020 to June 2021 and including seven articles which are summarized here below.

The special section is inspired by the fast-paced advances in wearable and point-of-care technologies of the last two decades. Devices such as smart watches, wearable heart rate monitors, and wearable blood pressure monitors are empowering individuals to not only meet fitness progress benchmarks but also achieve health and wellbeing goals. Point-of-care devices are becoming accepted and integrated within the medical establishment, with an increase driven by the pervasiveness of chronic and infectious diseases^1^ as well as the need of patient-centric care. Industry is playing a phenomenal role in this growth. The point-of-care diagnostic market size was 34.49 billion dollars in 2020 and experienced a COVID-19-related growth of 73.5% in the same year. It is expected to reach 81.37 billion in 2028.^1^ Restraining factors in this growth are regulatory policies that have at times slowed down, if not interrupted, the introduction of these novel medical devices. In that respect, there are promising signs from the U.S. Food and Drug Administration (FDA): the Emergency Use Authorizations (EUAs)^2^ used during the pandemic for both treatment and point of care system were essential in bringing both care and diagnostic in period of emergency, and the agency was also quick in revoking issued EUA when new evidence arose.^3^ Furthermore, the creation of the Digital Health Center of Excellence as an evolution of the Digital Health Program in the Center for Devices and Radiological Health shows an effort to “reimagine the FDA’s approach to ensuring all Americans have timely access to high-quality, safe, and effective digital health products” (see Ref. 4).

In his prospective, “Perspective on the increasing role of optical wearables and remote patient monitoring in the COVID-19 era and beyond,” Roblyer reports on the technological acceleration that the COVID pandemic has created in the context of optical wearable and point of care. His report outlines improvements in adoption of remote patient monitoring, wearables, and telehealth applications during the pandemic and beyond. Roblyer’s work provides an excellent summary of both optically derived parameters currently in use in remote care, and parameters that are most sought-after but remain to be developed.

Several of the articles submitted to the special section focused on improvements on methodologies for tissue oxygenation and perfusion that could be used in the management of a variety of conditions, including COVID-related illnesses.

Belcastro et al. (URL: https://doi.org/10.1117/1.JBO.25.8.082702) proposed a new design for a compact low-cost RGB spectral frequency domain imager that utilizes a color CMOS camera and LED-based miniprojector. This study was motivated by the need for noninvasive and inexpensive imagers capable of achieving both structural and chemical information of living tissue. Such system can be operated by lay individuals in remote settings. This work focuses on SFDI, a modality capable of separating the effects of scattering and absorption, which requires the spatial modulation of the source at specific frequencies. In the past, the use of a color imager and white light projector has limited the application to three spectral bands (RGB); the work by Belcastro et al. extends the spectral capability to two more spectral bands through a calibration procedure based on a separate spectrometer as well as camera spectral sensitivity characterization through a grating and white light illuminator. The approach was validated with well characterized optical phantoms and *in vivo* on the skin of a volunteer.

Liu et al. (URL: https://doi.org/10.1117/1.JBO.26.1.012705) developed a wearable fiber-free dual-wavelength diffuse speckle contrast flow oximetry system (DSCFO) for simultaneous measurements of blood flow and oxygenation variations in deep tissues. Alterations in deep tissue hemodynamics are the hallmark of a variety of pathological conditions such as cerebral hypoxia, septic shock, peripheral artery disease, and even tumors, to name a few. The authors propose an extension of their previous work on single-wavelength diffuse speckle contrast flowmetry (DSCF) technique to diffuse speckle contrast flow oximetry (DSCFO), by adding a second wavelength sensitive to oxygenated hemoglobin to their system. Their apparatus is low cost and compact, consisting of two laser diodes and a small CMOS camera. The use of the camera improves the sampling rate while reducing the cost and dimension of the probe. The authors utilize optical phantoms to demonstrate their system sensitivity and high signal-to-noise ratio (SNR) as well as hemodynamic changes during artery cuff occlusion in five individuals.

Koenig et al. (URL: https://doi.org/10.1117/1.JBO.26.1.012706) proposed a low-cost and wearable CMOS-based device for high-resolution spatial diffuse reflectance imaging of skin conditions. This system improves on current spatially resolved diffuse reflectance spectroscopy (srDRS) systems by not requiring expensive instrumentation, and fiber optics. This approach circumvents the limitations of short source-detector separation by utilizing a fiber optic plate. Three light emitting diodes (LEDs) were used at 511, 615, and 660 nm. The system is controlled by a custom circuit board connected to a computer. The novel apparatus performance was compared to off-the-shelf clinical devices: an StO2 sensor and a PtCo2 sensor. Measurements were conducted on 20 volunteers and generally good agreement was found between the low cost and commercial-grade devices (5% to 10% discrepancy in StO2 characterization was found depending on the analysis method), although two separate analysis methods were necessary at this stage. The authors point out the sensitivity of their system to skin tone, which is alleviated by the use of skin-matched (dark, light) reference samples.

Pai et al. (URL: https://doi.org/10.1117/1.JBO.26.2.022707) proposed a non-contact heart rate variability (HRV) algorithm utilizing common cameras. Remote and noncontact estimation of heart rate variability has many applications. These measurements commonly suffer from low SNR, due to the absorption properties of skin as well as unpredictable illumination and movement artifacts. The author’s approach improves on current methods by utilizing an automatic adaptive bandwidth filtering and discrete energy separation to estimate the instantaneous frequency of an image photoplethysmography signal. Both the algorithm and data set of 12 individuals utilized in this study are made available.

Implantable devices are becoming more popular as a way to manage chronic disease. A successful example of this application are glucose monitors which are implanted in the patient subcutis and interfaced to wearable systems for data monitoring and analysis. Cao et al. (URL: https://doi.org/10.1117/1.JBO.25.11.112704) proposed a new application for implantable sensors: the measurement of tissue oxygenation during radiotherapy in combination with Cherenkov-excited luminescence imaging (CELI). Studies have shown that oxygen enhances the radiobiologic damage improving cancer treatment outcome while the opposite has been shown in hypoxic environments. The sensor consists of an agarose matrix with a PtG4, an oxygen quenched dendritic molecule. It was tested both in phantoms and in an *in-vivo* tumor model undergoing fractionated radiotherapy.

Finally, Spink et al. (URL: https://doi.org/10.1117/1.JBO.26.6.062708) demonstrated the design and realization of a high optode-density wearable continuous wave diffuse optical probe for the monitoring of breathing hemodynamics in breast tissue. This work is motivated by the need to predict neoadjuvant chemotherapy (NAC) treatment response for locally advanced breast cancer patients. The probe was fabricated on a flexible 6 cm x 6 cm printed circuit board populated with 32 LED (750 and 850 nm) and 16 optical silicon detectors. Validation was conducted with both optical phantoms and *in vivo* with a cuff occlusion experiment as well as in a breast study of healthy volunteers.

The future is bright for research related to wearable and point-of-care technology. As illustrated by the investigators participating in this special section, optical science is bound to play a fundamental role in the advancement of integration in standard of care. We thank all the contributors and JBO editorial and production staff for their assistance.
